# Quantification of the margin required for treating intraprostatic lesions

**DOI:** 10.1120/jacmp.v17i3.6089

**Published:** 2016-05-08

**Authors:** Matthew T. Studenski, Yanisley Valenciaga, Matthew C. Abramowitz, Radka Stoyanova, Elizabeth Bossart, Nesrin Dogan, Alan Pollack

**Affiliations:** ^1^ Department of Radiation Oncology University of Miami Miller School of Medicine Miami FL USA; ^2^ Department of Biomedical Physics University of California Los Angeles Los Angeles CA USA

**Keywords:** MRI, prostate radiation therapy, intraprostatic lesion, IMRT

## Abstract

Advances in magnetic resonance imaging (MRI) sequences allow physicians to define the dominant intraprostatic lesion (IPL) in prostate radiation therapy treatments allowing for dose escalation and potentially increased tumor control. This work quantifies the margin required around the MRI‐defined IPL accounting for both prostate motion and deformation. Ten patients treated with a simultaneous integrated intraprostatic boost (SIIB) were retrospectively selected and replanned with incremental 1 mm margins from 0‐5 mm around the IPL to determine if there were any significant differences in dosimetric parameters. Sensitivity analysis was then performed accounting for random and systematic uncertainties in both prostate motion and deformation to ensure adequate dose was delivered to the IPL. Prostate deformation was assessed using daily CBCT imaging and implanted fiducial markers. The average IPL volume without margin was 2.3% of the PTV volume and increased to 11.8% with a 5 mm margin. Despite these changes in volume, the only statistically significant dosimetric difference was found for the PTV maximum dose, which increased with increasing margin. The sensitivity analysis demonstrated that a 3.0 mm margin ensures >95% IPL coverage accounting for both motion and deformation. We found that a margin of 3.0 mm around the MRI defined IPL is sufficient to account for random and systematic errors in IPL position for the majority of cases.

PACS number(s): 87.55.de

## I. INTRODUCTION

The use of special acquisition protocols with magnetic resonance imaging (MRI), such as dynamic contrast‐enhanced (DCE) imaging and diffusion‐weighted imaging (DWI), allow for functional information to be included along with anatomical information. One disease site in radiation oncology that has seen an increase in MRI is the prostate, where the high‐risk intraprostatic lesion (IPL) can be identified.^1^, ^2^, ^3^, ^4^, ^5^, ^6^, ^7^, ^8^, ^9^, ^10^, ^11^ Since dose escalation can improve cure rates, delivering an escalated dose to the IPL while treating the remainder of the prostate to the normal prescription dose can reduce toxicity while increasing tumor control.^12^, ^13^, ^14^ Technologies like intensity‐modulated radiation therapy (IMRT) allow for delivery of different dose levels using a simultaneous integrated intraprostatic boost (SIIB), but at the same time generate steep dose gradients requiring exact knowledge of the location of the prostate and IPL.

Four types of motion can affect the position of the prostate and IPL: inter‐ and intrafraction motion and inter‐ and intrafraction deformation.^(15)^ Intrafraction deformation is usually ignored as it is minimal during the delivery time.^(16)^ Interfraction motion can be mitigated using daily image guidance and intrafraction prostate motion has been studied using various tracking methods.^17^, ^18^ Standard prostate radiotherapy accounts for these inter‐ and intrafraction motions by including a 3‐8 mm margin around the prostate to create the planning target volume (PTV). These margins are well established, and account for the random and systematic errors involved in setting up the patient and prostate motion on a daily basis.^17^, ^19^, ^20^, ^21^

Interfraction deformation of the prostate poses a difficult problem, especially when there is a specific region of the prostate receiving an escalated dose. Deformation of the prostate would be contained in the PTV margin, but the IPL could shift out of the high‐dose region, reducing the benefit of dose escalation. Adding a margin to the IPL would reduce uncertainties in treatment delivery, but it could also lead to toxicity. Due to these competing interests, there is no consensus for the IPL margin to ensure adequate dose is delivered. In some recent studies the IPL is treated with no additional margin,^(5,8,22^) while in others margins of up to 8 mm are added.^2^, ^3^, ^4^, ^6^, ^7^, ^9^, ^10^, ^11^

One goal of this study is to evaluate the dosimetric impact on the surrounding organs at risk (OARs) when a margin is added to the IPL. A second goal is to assess the sensitivity of IPL coverage to random and systematic errors, including both prostate motion and deformation. Prostate motion is well understood, but prostate deformation on a daily basis has not been studied as thoroughly. To our knowledge, a study addressing all of these factors has not been done.

## II. MATERIALS AND METHODS

### A. Dosimetric study

Ten random patients were retrospectively selected from a group treated with a SIIB on an institutional review board (IRB)‐approved clinical trial focusing on image‐guided radiation therapy for prostate cancer. The IPL was defined by the attending radiation oncologist as the hypervascular region on DCE‐MRI, except for anterior lesions, which must also have restricted water motion on DWI. To reduce the uncertainty in delineating the IPL, semi‐automated software was developed to assist in this process.^(23)^ Extra care was taken while defining the IPL near the rectum to avoid unnecessary toxicity.

As a note, in our department, we follow a strict protocol for bladder and rectal filling. If during the initial CBCT the bladder is not full or the rectum is too full, the patient will be taken down from the table and either given water to fill the bladder or allowed to void their rectum before being set up again. This method is used for all prostate and prostate bed patients, so it is important to realize the results presented in this paper are affected by this method.

Two 358°, 10 MV RapidArcs (Varian Medical Systems, Palo Alto, CA) were used to deliver 76 Gy to the PTV and 89.3 Gy to the IPL in 38 fractions. Assuming an α/β ratio of 3.0 Gy, this fractionation scheme delivers a 2.0 Gy equivalent dose of 95.5 Gy to the GTV. Each plan was calculated in the Eclipse treatment planning system (Varian) using the Acuros algorithm (dose to medium), version 11.0.31.

The original treatment plans did not have a margin around the IPL so each case was replanned by a single physicist with incremental 1 mm margins from 0‐5 mm around the IPL. To avoid confusion throughout the paper, the IPL with margin will be referred to as ${\text{PTV}}_{I}$ and the prostate with margin will be referred to as ${\text{PTV}}_{P}$. The ${\text{PTV}}_{P}$ is the standard PTV treated for all prostate cancer patients at our institution. All plans were optimized from scratch. The ${\text{PTV}}_{I}$ was cropped if it extended beyond the ${\text{PTV}}_{P}$ but it was allowed to impinge upon OARs. Per clinical protocol, the plans were scaled so the prescription dose coverage was maintained above 95% for the ${\text{PTV}}_{P}$ and ${\text{PTV}}_{I}$. The volume of the rectum receiving 65 Gy and 40 Gy was kept below 17% and 35%, respectively, and the maximum dose was kept below 85.5 Gy. The volume of the bladder receiving 65 Gy and 40 Gy was kept below 25% and 50%, respectively.

For the dosimetric analysis, ${\text{PTV}}_{P}$ D95% and maximum dose, rectum V65 Gy, V40 Gy and D1cc, and bladder V65 Gy and V40 Gy were obtained from the dose‐volume histogram (DVH). We used paired, two‐tailed *t*‐tests to determine statistical significance (p<0.05) in dosimetric parameters.

### B. Prostate deformation

Deformation induces systematic ($\sum_{\text{deform}}$) and random ($\sigma_{\text{deform}}$) uncertainty in the daily delivery. A systematic deformation would be a result of an inconsistent practice such as not monitoring bladder and rectal filling on a daily basis leading to deformation from the planning volume. A random deformation would be a more transient effect such as rectal gas causing an unpredicted deformation. In addition, there are well‐established systematic ($\sum_{\text{setup}}$) and random ($\sigma_{\text{setup}}$) errors in patient setup and prostate motion that must be accounted for during planning and treatment delivery. Values for $\sum_{\text{setup}}$ and $\sigma_{\text{setup}}$ have been previously published using similar daily image guidance (without an endorectal balloon).^20^, ^24^, ^25^ Taking an average of these studies, $\sum_{\text{setup}}$ was 0.82 mm, 0.97 mm, and 1.19 mm in the LR, AP, and SI directions, respectively, and $\sigma_{\text{setup}}$ was 1.46 mm, 2.14 mm, and 1.74 mm in the LR, AP, and SI directions, respectively.

These studies did not account for interfraction deformation, so we needed to determine $\sum_{\text{deform}}$ and $\sigma_{\text{deform}}$ separately. Fifteen sequential patients treated for prostate cancer at our institution were retrospectively selected. Fourteen patients had four and one patient had three implanted markers. All received a dose of 80 Gy in 40 fractions with daily cone‐beam CT (CBCT). CBCT image sets were acquired with a 2.5 mm slice thickness using the pelvis spotlight protocol on the Varian OBI system. The 40 CBCT image sets for each patient were segmented using an in‐house MATLAB (MathWorks, Inc., Natwick, MA) routine that identified the centroid of each fiducial marker. The distance between the centroids in three dimensions was calculated for each individual marker combination. Eighty‐seven marker combinations were analyzed: six combinations for the patients with four markers and three combinations for the patient with three markers.

The mean distance for each marker combination (e.g., Patient 1, markers 1 and 3) was calculated over the 40 fractions. The mean of the 40 distances was defined as the true distance and the daily deviation from this true distance was calculated in the left‐right, superior‐inferior, and anterior‐posterior directions. The total three‐dimensional distance between the markers was also obtained. The mean distance was chosen as the true distance to mitigate the known effect of fiducial migration.^(19,26^) Choosing the true distance from the planning CT or Day 1 CBCT could bias the results and lead to artificially larger deformations during the final fractions. This issue is addressed in more detail in the discussion section.

For each patient, there were 40 deviations from the true distance. The mean (M) of these deviations represented the systematic prostate deformation, while the standard deviation (SD) represented the random deformation. To calculate $\sum_{\text{deform}}$ for all 87 marker combinations, the standard deviation of all of the mean daily deviations (SD of all Ms) was obtained. The $\sigma_{\text{deform}}$ for all of the marker combinations was determined by calculating the root mean square (RMS) of all of the daily standard deviations (RMS of all SDs). The total systematic and random error was calculated in each dimension using the following two equations:

(1) $$\sum_{total}^{2}=\sum_{setup}^{2}+\sum_{deform}^{2}$$

(2) $$\sigma_{total}^{2}=\sigma_{setup}^{2}+\sigma_{deform}^{2}$$

### C. Sensitivity analysis

The 10 patients from the dosimetric study (60 plans total) were selected to analyze the sensitivity of IPL coverage to random and systematic errors. We used the plan robustness analysis tool in the Computational Environment for Radiotherapy Research (CERR) software.^(27)^ This tool uses the dose distribution, number of fractions, and three‐dimensional random and systematic errors as input to generate a DVH with upper and lower bounds simulating the errors during delivery for an entire course of treatment. One hundred iterations were run in the plan robustness analysis tool for each dose distribution (0 mm margin up to a 5 mm margin), and the volume of the ${\text{PTV}}_{I}$ receiving more than 89.3 Gy (${\text{PTV}}_{I}$ V89.3 Gy) was recorded along with the 1‐sigma bounds.

## III. RESULTS

### A. Dosimetric study

For all the patients, the average IPL volume was 4.1 cc (2.3% of the ${\text{PTV}}_{P}$ volume). When the margin was increased to 5 mm, the average ${\text{PTV}}_{I}$ volume increased to 20.2 cc (11.8% of the ${\text{PTV}}_{P}$ volume). ${\text{PTV}}_{I}$ volumes ranged from 0.9% of the ${\text{PTV}}_{P}$ volume up to 22.2%, as seen in Table 1.

**Figure 1:**
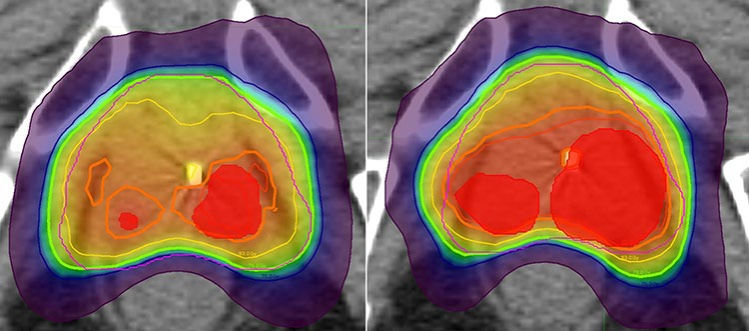
Representative axial slice showing the dose distribution for one patient. The left shows the ${\text{PTV}}_{I}$ (shaded red) with 0 mm margin and the right with a 5 mm margin. The thick orange isodose line is 89.3 Gy and the thick green isodose line is 76 Gy. Note that with the 5 mm margin the 89.3 Gy isodose line is pushed away from the rectum, requiring a hotter plan to achieve adequate ${\text{PTV}}_{I}$ coverage.

In all cases, the clinical protocol constraints were met. The only statistically significant difference found was for the ${\text{PTV}}_{P}$ maximum dose, which increased steadily from 93.6±1.0 Gy in plans with no margin up to 95.9±2.2 Gy with a 5 mm margin (p=0.0088). No statistical differences were seen for any other parameter and a larger margin did not necessarily correlate with a higher dose to OARs, as seen in Table 2. A contributing factor to these results can be seen in Fig. 1, which shows a representative slice from one patient demonstrating the difference in dose distribution for a ${\text{PTV}}_{I}$ with no margin and with a 5 mm margin. The optimizer only knows to meet the applied constraints and in cases with ${\text{PTV}}_{I}\;\backslash \text{rectal}$ overlap, the optimizer struggles to find a solution to satisfy the maximum rectal dose constraint. The optimizer can only push the hot spot away from the rectum, which in turn requires a hotter plan to achieve the 95% dose coverage of the ${\text{PTV}}_{I}$ Additionally, the hot spots in the ${\text{PTV}}_{P}$ are not necessarily linked and can end up in random locations inside the ${\text{PTV}}_{P}$ as long as the optimizer achieves the 95% coverage. This leads to the lack of margin‐to‐dose correlation seen in Table 2.

**Table 1:** ${\text{PTV}}_{I}$ volume as a percentage of ${\text{PTV}}_{P}$ volume for the 10 patients in the dosimetric study.

*Margin*	*0 mm*	*1 mm*	*2 mm*	*3 mm*	*4 mm*	*5 mm*
Pat. 1	4.3	8.9	8.9	11.8	13.2	16.3
Pat. 2	3.3	5.6	7.1	8.3	10.6	12.5
Pat. 3	6.7	11.3	13.9	15.9	19.4	22.2
Pat. 4	1.2	3.2	4.8	6.0	9.1	11.4
Pat. 5	2.2	4.1	5.3	6.4	8.5	10.3
Pat. 6	0.7	2.6	4.0	5.0	6.9	8.4
Pat. 7	1.8	4.3	5.7	7.3	9.8	11.8
Pat. 8	1.5	3.6	5.0	5.9	8.0	9.5
Pat. 9	1.2	3.7	5.6	7.1	10.3	12.6
Pat. 10	0.3	0.9	1.4	1.7	2.7	3.4
Average	2.3	4.8	6.2	7.5	9.8	11.8

**Table 2:** Dosimetric OAR results for the 10 patients (p‐values show significance compared to 0 mm margin).

	*Bladder V65 Gy %*		*Bladder V40 Gy (%)*		*Rectum V65 Gy (%)*		*Rectum V40 Gy (%)*		*Rectum D1cc (cGy)*	
*Margin (mm)*	*Mean*	*p‐value*	*Mean*	*p‐value*	*Mean*	*p‐value*	*Mean*	*p‐value*	*Mean*	*p‐value*
0	13.5	N/A	33.1	N/A	9.3	N/A	27.1	N/A	7666.6	N/A
1	13.4	0.441	33.3	0.745	8.7	0.140	26.2	0.139	7654.1	0.700
2	13.3	0.486	32.4	0.440	8.5	0.101	25.4	0.067	7648.3	0.817
3	13.4	0.709	32.6	0.593	8.6	0.226	25.4	0.083	7658.0	0.881
4	13.6	0.618	32.3	0.370	9.0	0.670	26.0	0.323	7732.4	0.461
5	13.8	0.178	32.6	0.553	9.2	0.918	26.1	0.371	7794.3	0.269

### B. Prostate deformation

**Figure 2:**
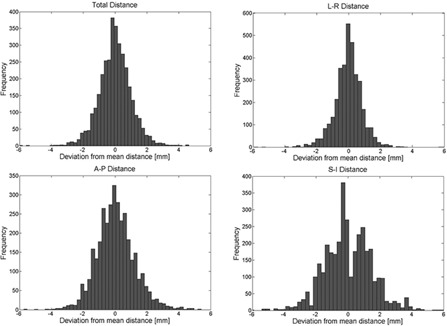
Distribution of the daily deviations from the true distance for all marker positions.

Figure 2 shows the distribution of the marker deviations from the true distance. Seventy‐three percent (73%) of the deviations were less than 1.0 mm and only 0.9% were greater than 3.0 mm. The average standard deviation (SD) from the true distance for all patients was 0.95 mm (0.14 mm up to 2.50 mm).

The average standard deviation from the true distance in the LR direction was 0.76 mm (maximum of 1.79 mm), while the average for the AP direction was 1.14 mm (maximum of 2.04 mm) and in the SI direction it was 1.39 mm (maximum of 2.53 mm). The maximum absolute distance deviation was 6.11 mm in the LR direction, 5.40 mm in the AP direction, and 6.28 mm in the SI direction. Most deformation occurs in the AP or SI direction, which can be attributed to variable rectal and bladder filling.

$\sum_{\text{deform}}$ and $\sigma_{\text{deform}}$ were calculated using the results of the deformation study. $\sum_{\text{deform}}$ was 0.21 mm, 0.26 mm, and 0.40 mm in the LR, AP, and SI directions, respectively; $\sigma_{\text{deform}}$ was 0.5 mm, 0.72 mm, and 0.79 mm in the LR, AP, and SI directions, respectively.

### C. Sensitivity analysis

Using Eq. (1) and (2) we calculated that $\sum_{\text{total}}$ was 0.85 mm, 1.10 mm, and 1.19 mm and $\sigma_{\text{total}}$ was 1.46 mm, 2.14 mm, and 1.74 mm in the LR, AP, and SI directions, respectively. Table 3 shows the results from the sensitivity analysis for the ${\text{PTV}}_{I}$ V89.3 Gy. The mean, standard deviation (SD), and range for the 10 patients for each parameter are reported. With no margin, the ${\text{PTV}}_{I}$ V89.3 Gy is on average 84.7%, below the clinical constraint of 95%. As expected, when the margin increased, the ${\text{PTV}}_{I}$ V89.3 Gy increased. With a margin of 3 mm, all patients achieved ${\text{PTV}}_{I}$ V89.3 Gy coverage of >95%.

**Table 3:** Results for ${\text{PTV}}_{I}$ V89.3 Gy from the sensitivity analysis in CERR. The mean, standard deviation (SD), and range for the 10 patients are presented.

	$PTV_{I}\;V89.3\;Gy$ *%*			*Lower Bound* (1σ) *%*			*Upper Bound* (1σ) *%*		
*Margin (mm)*	*Mean*	*SD*	*Range*	*Mean*	*SD*	*Range*	*Mean*	*SD*	*Range*
0	84.7	6.7	(72.9‐93.2)	80.5	8.0	(66.4‐91.6)	90.3	4.2	(84.8‐95.8)
1	94.7	1.9	(92.4‐97.9)	91.5	3.1	(88.3‐96.3)	98.5	0.9	(97.1‐100)
2	96.6	2.0	(91.7‐98.7)	93.5	3.3	(85.6‐97.6)	99.7	0.4	(98.8‐100)
3	98.3	0.7	(97.0‐99.4)	96.3	1.5	(94.3‐98.5)	99.9	0.2	(99.4‐100)
4	99.2	0.8	(97.2‐99.9)	98.2	1.4	(94.8‐99.7)	99.9	0.2	(99.5‐100)
5	99.4	0.7	(97.6‐99.9)	98.7	1.5	(94.8‐ 99.8)	99.9	0.1	(99.7‐100)

## IV. DISCUSSION

Other studies have looked at inter‐ and intrafraction motion of the prostate, but all of them assume that the interfraction deformation is minimal or dosimetrically irrelevant.^15^, ^16^, ^17^, ^21^, ^28^ For standard cases, this assumption holds true, but when a particular area of the prostate is receiving an escalated dose, interfraction deformation can influence the tumor control and the normal tissue complications. Additionally, there is a distinct lack of consensus between studies on the appropriate margin that should be used for the IPL. Our study is novel in that it is the only one to present a dosimetric analysis, an analysis of intrafraction deformations, and a sensitivity analysis to quantify the IPL margin.

For some of the studies that include a simultaneous boost, no margin was provided for the IPL assuming that interfraction deformation is minimal.^5^, ^8^, ^11^ Other studies included a margin up to 8 mm around the IPL but not because of prostate deformation. Onal et al.^(7)^ and Pinkawa et al.^(9)^ used a 3‐4 mm margin to account for intrafraction motion. Housri et al.^(3)^ and Miralbell et al.^(6)^ used an isotropic 3 mm margin, although the Miralbell study treated with a rectal balloon (limited central mass displacement to less than 3 mm), while Housri and colleagues used cine‐MRI, ultrasound, and fiducial marker tracking to determine that the intrafraction displacement was less than 3 mm. Singh et al.^(11)^ used a 3 mm margin due to limitations in image guidance. Fonteyne et al.^(2)^ used an 8 mm margin to account for planning uncertainties. Ippoliot et al.^(4)^ and Riches et al.^(10)^ do not provide an explanation of their choices of 5 mm and 2 mm margins, respectively. Our study agrees with many of these studies with the magnitude of the IPL margin, but here, the result is supported by a dosimetric study, deformation study, and sensitivity analysis.

The major limitation in this study is the inability to visualize the IPL on the daily CBCT imaging. Daily MRI would be ideal as the IPL motion, deformation, shape, and tumor biology changes could be evaluated to calculate the margin but this technology is not available in our department. Instead, fiducial markers were used as a surrogate since they were readily seen on the daily CBCT images. Nichol et al.^(19)^ considered a similar technique but their study was limited as only two image sets were acquired over the entire treatment course.

**Figure 3:**
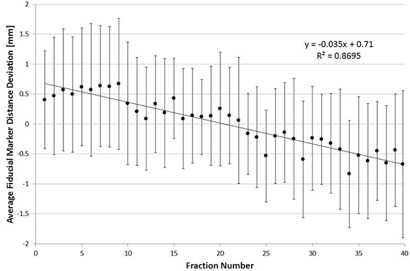
Average of the 87 fiducial marker distance deviations vs. fraction number. The error bars represent ±1 SD.

Another confounding effect in this current study is fiducial marker migration. Shirato et al.^(26)^ and Nichol and colleagues^(19)^ found a significant in‐migration of fiducial markers of 0.05 mm/fraction, while Dehnad et al.^(29)^ and Kupelian et al.^(30)^ concluded that fiducial migration was minimal. We plotted the average fiducial marker deviation from the true distance over the treatment course (Fig. 3) and we found an in‐migration of about 0.03 mm/fraction (1.4 mm total). The effect of this in‐migration was mitigated in this work by assuming the true marker distance was an average of all of the daily distances and then using the absolute value of the deviation to calculate $\sum_{\text{deform}}$ and $\sigma_{\text{deform}}$.

## V. CONCLUSIONS

The benefit of dose escalation is only applicable if the dose is delivered to the correct location. The results presented here show that a margin of 3.0 mm around the IPL is sufficient to account for prostate motion and deformation to ensure that the IPL is receiving the escalated dose. This margin should not present any dosimetric treatment planning problems.

## ACKNOWLEDGMENTS

This work was supported in part by Grant 10BN03 from the Bankhead Coley Cancer Research Program and was presented in part as an abstract at the 2014 AAPM annual meeting (SU‐F‐BRD‐06).

## COPYRIGHT

This work is licensed under a Creative Commons Attribution 4.0 International License.

## References

[1] Bauman G , Haider M , van der Heide UA , Ménard C . Boosting imaging defined dominant prostatic tumors: a systematic review. Radiother Oncol. 2013;107(3):274–81. 23791306 10.1016/j.radonc.2013.04.027

[2] Fonteyne V , Villeirs G , Speleers B , et al. Intensity‐modulated radiotherapy as primary therapy for prostate cancer: report on acute toxicity after dose escalation with simultaneous integrated boost to intraprostatic lesion. Int J Radiat Oncol Biol Phys. 2008;72(3):799–807. 18407430 10.1016/j.ijrobp.2008.01.040

[3] Housri N , Ning H , Ondos J , Choyke P , Camphausen K , Citrin D , Arora B , Shankavaram U , Kaushal A . Parameters favorable to intraprostatic radiation dose escalation in men with localized prostate cancer. Int J Radiat Oncol Biol Phys. 2011;80:614–20. 20932672 10.1016/j.ijrobp.2010.06.050 PMC3580994

[4] Ippolito E , Mantini G , Morganti AG , et al. Intensity‐modulated radiotherapy with simultaneous integrated boost to dominant intraprostatic lesion: preliminary report on toxicity. Am J Clin Oncol. 2012;35(2):158–62. 21336090 10.1097/COC.0b013e318209cd8f

[5] De Meerleer GD , Villeirs G , Bral S , et al. The magnetic resonance detected intraprostatic lesion in prostate cancer: planning and delivery of intensity‐modulated radiotherapy. Radiother Oncol. 2005;75(3):325–33. 15967524 10.1016/j.radonc.2005.04.014

[6] Miralbell R , Mollà M , Rouzaud M , et al. Hypofractionated boost to the dominant tumor region with intensity modulated stereotactic radiotherapy for prostate cancer: a sequential dose escalation pilot study. Int J Radiat Oncol Biol Phys. 2010;78(1):50–57. 19910135 10.1016/j.ijrobp.2009.07.1689

[7] Onal C , Sonmez S , Erbay G , Guler OC , Arslan G . Simultaneous integrated boost to intraprostatic lesions using different energy levels of intensity‐modulated radiotherapy and volumetric‐arc therapy. Br J Radiol. 2014;87(1034):20130617. 24319009 10.1259/bjr.20130617 PMC4064542

[8] Ost P , Speleers B , De Meerleer G , et al. Volumetric arc therapy and intensity‐modulated radiotherapy for primary prostate radiotherapy with simultaneous integrated boost to intraprostatic lesion with 6 and 18 MV: a planning comparison study. Int J Radiat Oncol Biol Phys. 2011;79(3):920–26. 20675077 10.1016/j.ijrobp.2010.04.025

[9] Pinkawa M , Attieh C , Piroth MD , et al. Dose‐escalation using intensity‐modulated radiotherapy for prostate cancer — evaluation of the dose distribution with and without 18F‐choline PET‐CT detected simultaneous integrated boost. Radiother Oncol. 2009;93(2):213–19. 19717197 10.1016/j.radonc.2009.07.014

[10] Riches S , Payne GS , Desouza NM , et al. Effect on therapeutic ratio of planning a boosted radiotherapy dose to the dominant intraprostatic tumour lesion within the prostate based on multifunctional MR parameters. Br J Radiol. 2014;87(1037):20130813. 24601648 10.1259/bjr.20130813 PMC4075537

[11] Singh AK , Guion P , Sears‐Crouse N , et al. Simultaneous integrated boost of biopsy proven, MRI defined dominant intra‐prostatic lesions to 95 Gray with IMRT: early results of a phase I NCI study. Radiat Oncol. 2007;2:36. 17877821 10.1186/1748-717X-2-36 PMC2075521

[12] Pollack A , Zagars GK , Starkschall G , et al. Prostate cancer radiation dose response: results of the M. D. Anderson phase III randomized trial. Int J Radiat Oncol Biol Phys. 2002;53(5):1097–105. 12128107 10.1016/s0360-3016(02)02829-8

[13] Zietman AL , DeSilvio ML , Slater JD , et al. Comparison of conventional‐dose vs high‐dose conformal radiation therapy in clinically localized adenocarcinoma of the prostate: a randomized controlled trial. JAMA. 2005;294(10):1233–39. 16160131 10.1001/jama.294.10.1233

[14] Zelefsky MJ , Yamada Y , Fuks Z , et al. Long‐term results of conformal radiotherapy for prostate cancer: impact of dose escalation on biochemical tumor control and distant metastases‐free survival outcomes. Int J Radiat Oncol Biol Phys. 2008;71(4):1028–33. 18280056 10.1016/j.ijrobp.2007.11.066

[15] Kupelian PA , Langen KM , Willoughby TR , Zeidan OA , Meeks SL . Image‐guided radiotherapy for localized prostate cancer: treating a moving target. Semin Radiat Oncol. 2008;18(1):58–66. 18082589 10.1016/j.semradonc.2007.09.008

[16] Tehrani JN , O'Brien RT , Poulsen PR , Keall P . Real‐time estimation of prostate tumor rotation and translation with a kV imaging system based on an iterative closest point algorithm. Phys Med Biol. 2013;58(23):8517–33. 24240537 10.1088/0031-9155/58/23/8517

[17] Kupelian P , Willoughby T , Mahadevan A , et al. Multi‐institutional clinical experience with the Calypso System in localization and continuous, real‐time monitoring of the prostate gland during external radiotherapy. Int J Radiat Oncol Biol Phys. 2007;67(4):1088–98. 17187940 10.1016/j.ijrobp.2006.10.026

[18] Both S , Wang KK‐H , Plastaras JP , et al. Real‐time study of prostate intrafraction motion during external beam radiotherapy with daily endorectal balloon. Int J Radiat Oncol Biol Phys. 2011;81(5):1302–09. 21035952 10.1016/j.ijrobp.2010.08.052

[19] Nichol AM , Brock KK , Lockwood GA , et al. A magnetic resonance imaging study of prostate deformation relative to implanted gold fiducial markers. Int J Radiat Oncol Biol Phys. 2007;67(1):48–56. 17084546 10.1016/j.ijrobp.2006.08.021

[20] Beltran C , Herman MG , Davis BJ . Planning target margin calculations for prostate radiotherapy based on intrafraction and interfraction motion using four localization methods. Int J Radiat Oncol Biol Phys. 2008;70(1):289–95. 17919837 10.1016/j.ijrobp.2007.08.040

[21] Litzenberg DW , Balter JM , Hadley SW , et al. Influence of intrafraction motion on margins for prostate radiotherapy. Int J Radiat Oncol Biol Phys. 2006;65(2):548–53. 16545919 10.1016/j.ijrobp.2005.12.033

[22] Wong WW , Schild SE , Vora SA , et al. Image‐guided radiotherapy for prostate cancer: a prospective trial of concomitant boost using indium‐111‐capromab pendetide (ProstaScint) imaging. Int J Radiat Oncol Biol Phys. 2011;81(4):e423–29. 21477947 10.1016/j.ijrobp.2011.01.048

[23] Stoyanova R , Sandler K , Pollack A . Delineation and visualization of prostate cancer in multiparametric MRI. Pract Radiat Oncol. 2012;3(2 Suppl 1):S30–S31. 10.1016/j.prro.2013.01.105 24674544

[24] Tanyi JA , He T , Summers PA , et al. Assessment of planning target volume margins for intensity‐modulated radiotherapy of the prostate gland: role of daily inter‐and intrafraction motion. Int J Radiat Oncol Biol Phys. 2010;78(5):1579–85. 20472357 10.1016/j.ijrobp.2010.02.001

[25] Shiraishi K , Futaguchi M , Haga A , et al. Validation of planning target volume margins by analyzing intrafractional localization errors for 14 prostate cancer patients based on three‐dimensional cross‐correlation between the prostate images of planning CT and intrafraction cone‐beam CT during volumetric modulated arc therapy. Biomed Res Int. 2014;2014:960928. 24977167 10.1155/2014/960928 PMC4055024

[26] Shirato H , Harada T , Harabayashi T , et al. Feasibility of insertion/implantation of 2.0‐mm‐diameter gold internal fiducial markers for precise setup and real‐time tumor tracking in radiotherapy. Int J Radiat Oncol Biol Phys. 2003;56(1):240–47. 12694845 10.1016/s0360-3016(03)00076-2

[27] Deasy JO , Blanco AI , Clark VH . CERR: a computational environment for radiotherapy research. Med Phys. 2003;30(5):979. 12773007 10.1118/1.1568978

[28] Abdellatif A , Craig J , Jensen M , et al. Experimental assessments of intrafractional prostate motion on sequential and simultaneous boost to a dominant intraprostatic lesion. Med Phys. 2012;39(3):1505–17. 22380383 10.1118/1.3685586

[29] Dehnad H , Nederveen AJ , van der Heide UA , van Moorselaar RJ , Hofman P , Lagendijk JJ . Clinical feasibility study for the use of implanted gold seeds in the prostate as reliable positioning markers during megavoltage irradiation. Radiother Oncol. 2003;67(3):295–302. 12865177 10.1016/s0167-8140(03)00078-1

[30] Kupelian PA , Willoughby TR , Meeks SL , et al. Intraprostatic fiducials for localization of the prostate gland: monitoring intermarker distances during radiation therapy to test for marker stability. Int J Radiat Oncol Biol Phys. 2005;62(5):1291–96. 16029784 10.1016/j.ijrobp.2005.01.005

